# Research progress of functional motifs based on growth factors in cartilage tissue engineering: A review

**DOI:** 10.3389/fbioe.2023.1127949

**Published:** 2023-02-07

**Authors:** Shengao Qin, Jiaman Zhu, Guangyong Zhang, Qijia Sui, Yimeng Niu, Weilong Ye, Guowu Ma, Huiying Liu

**Affiliations:** ^1^ School of Stomatology, Dalian Medical University, Dalian, China; ^2^ Academician Laboratory of Immune and Oral Development and Regeneration, Dalian Medical University, Dalian, China; ^3^ Beijing Key Laboratory of Tooth Regeneration and Function Reconstruction, Beijing Stomatological Hospital, School of Stomatology, Capital Medical University, Beijing, China; ^4^ Department of Stomatology, Beijing Friendship Hospital, Capital Medical University, Beijing, China

**Keywords:** cartilage tissue engineering, growth factors, functional motifs, mimetic peptides, chondrogenesis

## Abstract

Osteoarthritis is a chronic degenerative joint disease that exerts significant impacts on personal life quality, and cartilage tissue engineering is a practical treatment in clinical. Various growth factors are involved in cartilage regeneration and play important roles therein, which is the focus of current cartilage repair strategy. To compensate for the purification difficulty, high cost, poor metabolic stability, and circulating dilution of natural growth factors, the concept of functional motifs (also known as mimetic peptides) from original growth factor was introduced in recent studies. Here, we reviewed the selection mechanisms, biological functions, carrier scaffolds, and modification methods of growth factor-related functional motifs, and evaluated the repair performance in cartilage tissue engineering. Finally, the prospects of functional motifs in researches and clinical application were discussed.

## 1 Introduction

Various growth factors participate in the treatment of systemic diseases. Fibroblast growth factor-2 (FGF2) (Beenken and Mohammadi, 2009), transforming growth factor-β (TGF-β) (Maeda et al., 2013) and platelet-derived growth factor (PDGF) (Andrae et al., 2008) were reported to play key roles in the repair of periodontal defects. In addition, FGF2 does favor to repair skin wounds (Chan et al., 2017) and tracheal defects (Kitamura et al., 2011), promotes ligament regeneration (Kimura et al., 2008), and treats myocardial infarction (Itoh and Ornitz, 2011). Hematopoietic growth factor (HGF) was administrated in liver tissue regeneration and treatment of liver cirrhosis by binding to the c-Met receptor (Funakoshi and Nakamura, 2003). Applications of vascular endothelial growth factor (VEGF) (Hanft et al., 2008) and PDGF (Mulder et al., 2009) in the treatment of diabetic foot ulcers exhibited good clinical prospects, and ocular anti-VEGF for age-related macular changes showed significant advance in modern medicine (Cheung et al., 2014). In summary, various growth factors play active therapeutic roles in modern medicines and tissue engineering.

Chondral defects are important causes of osteoarthritis (OA) and joint disability in the elderly, and tissue engineering has been widely studied as a promising strategy (Williams et al., 2005). The three elements of cartilage tissue engineering are seed cells that initiate tissue reconstruction, biological scaffolds that provide support and guidance, and growth factors that induce chondrogenic differentiation of seed cells and cartilage matrix secretion. However, hyaluronic cartilage shows little potential for self-repair owing to lack of blood supply (Mascarenhas et al., 2015). Unlike bone regeneration and inflammation repair, growth factors cannot enrich effectively at local tissue through blood circulation (Jakobsen et al., 2005), which seriously affects the cartilage repair efficiency. Direct application of intact proteins is limited for easy degradation and dilution, and high cost of labor and finance during purification. Therefore, functional motifs were considered as alternatives of intact proteins.

Functional motifs are a series of short peptides, whose sequences originate from a specific growth factor, so as to simulate biological domains in amino acid sequences or microspatial structures. For example, Pierschbacher *et al.* found the sequence Arg-Gly-Asp (RGD) in fibronectin that binds to integrin (Pierschbacher et al., 1985). Then synthesized RGD can bind to the fibronectin receptor on the cell surface and promote cell adhesion, which does favor to survival of stem cells in cartilage regeneration. Cwirla *et al.* screened a peptide from the human thrombopoietin (TPO) receptor and obtained a 14-peptide sequence to mimic natural TPO *in vitro* (Dower et al., 1998).

By mature solid-phase synthesis technology (Behrendt et al., 2016), short peptides with fewer amino acid units have been widely applied in chondrogenesis studies for their simple operation and high production capacity, as well as advanced purification technology. In this paper, we focused on the selection mechanism, carrier scaffold, and modification methods of functional motifs, and summarized the application of functional motifs in cartilage tissue engineering *in vitro* and *in vivo*. Finally, the application prospect of functional motifs in cartilage repair is analyzed and discussed.

## 2 Mechanism of functional motif screening

**FIGURE 1:**
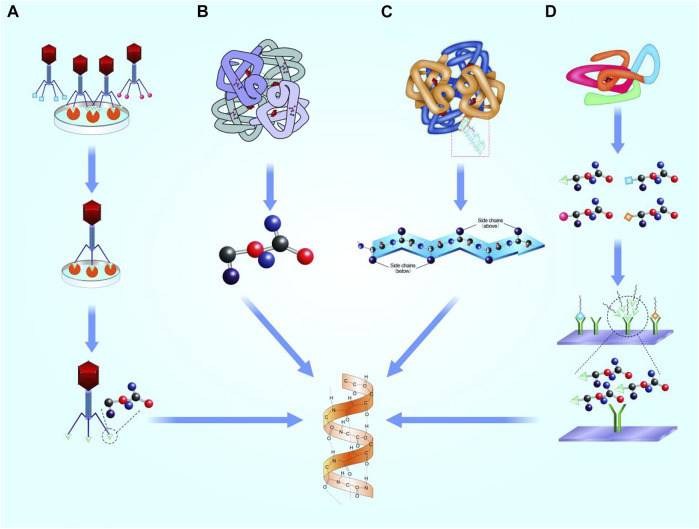
Selection mechanisms of functional motifs. **(A)**. Phage display technology. **(B)**. Highly repetitive conserved sequences. **(C)**. Microarray chip. **(D)**. Microstructure simulation of functional domain.

Interactions between proteins based on local domains of specific peptides. Geysen *et al.* proposed that short peptides containing key amino acid sequences can simulate certain bioactivity of proteins (Geysen et al., 1996). Banner *et al.* analyzed the crystal structure of human tumor necrosis factor (TNF) receptor and TNF-β complex, and found that only three clusters of amino acid residues acted between the ligand and its receptor, proving that only short peptides were involved in the interaction. Thus, it is possible to design functional motifs as mimetics of whole proteins (Banner et al., 1993). Generally, the screening mechanisms of motifs based on four concepts, as follows (Figure 1).

### 2.1 Phage display technology

Phage display technology relies on a peptide library to acquire functional motifs. An exogenous gene coding a short peptide was inserted into side chain gene of shell protein of a filamentous phage III (*p* III) or IV. A fusion protein attached to N-terminal of (*p* III) or IV that expressed on the shell surface of phages. Thus, specific lengths of phage aggregation of different sequences of exogenous peptides are presented, which constitute a good coverage peptide library (Smith and Scott, 1993). Phages were used to bind targets, and short peptides with highly affinity with targets were obtained after multiple screening. Finally, motifs were obtained by chemical synthesis.

In 1998, Cwirla *et al.* screened the phage peptide library through the human TPO receptor and obtained a 14-peptide sequence, which had similar activity with TPO *in vitro* (Dower et al., 1998). In the same year, Lowman *et al.* used insulin-like growth factors-binding protein (IGFBP) to obtain an insulin-like growth factors (IGF) related peptide motif. The acquired motif can bind to IGFBP and function in cartilage repair (Lowman et al., 1998). Subsequently, Ballinger *et al.* screened polypeptide C-19, which can mimic binding of basic fibroblast growth factor (bFGF) to the receptor and showed the same activity as FGF (Ballinger et al., 1999). Koishi *et al.* discovered a peptide sequence, HSNGLPL, with binding affinity to TGF-β1, indicating important influence on the formation of connective tissue (McLennan and Koishi, 2004). Gelain *et al.* screened PFSSTKT and SKPPGTSS, functional short peptides derived from bone marrow homing peptides (BMHP), to recruit stem cell for knee cartilage repair (Gelain et al., 2006). Akkiraju *et al.* screened and synthesized bone morphogenetic protein (BMP) functional motifs CK2.1 (Syed), CK2.2 (SLYD), and CK2.3 (SLKD). CK2.1 was the most promising peptide that induces chondrogenesis rather than osteogenesis (Akkiraju et al., 2017a). The limits of phage display technology were high cost of time and labor in phage preparation for screening motif peptides.

### 2.2 Highly repetitive conserved sequences

Highly repetitive sequences might exist in some proteins. Pierschbacher et al. (1985) found RGD sequence in fibronectin, collagen, and thrombin, which can bind to the fibronectin receptor on the cell surface and promote cell adhesion. In addition, Gelain *et al.* used amino acids K, *p*, F, S, and T to synthesize a series of short peptides with strong affinity to stem cells, including PFSSTKT and SKPPGTSS (Gelain et al., 2006). Williams et al. (2000) found neural cadherin (N-cadherin) has an evolutionarily conserved sequence, His-Ala-Val (HAV), which provides a homophile cell adhesion domain to mediate cell-cell adhesion (Blaschuk et al., 1990). Williams *et al.* performed a series of amino acid modifications on the HAV sequence, and found that acquired peptides showed similar binding ability to N-cadherin (Williams et al., 2001). Bian applied HAV in cartilage tissue engineering and thereby promoted the synthesis of cartilage matrix in rats model (Bian et al., 2013). However, some problems remain alongside with this method. Firstly, not all peptides possess highly repetitive conserved sequences that could be recognized. Even if a series of motifs are chosen, the sequence summarization of motifs depends on the experience of researchers, and the bioactivity of motifs needs to be verified.

### 2.3 Microarray chip

For some proteins without highly repetitive sequences, microarray chip technology can be used to systematically screen specific sequences (Foong et al., 2012). In microarray chip assay, thousands of short molecules—such as DNA, peptides, small chemical molecules, and cells—are arrayed on a chip as receptor (Sun et al., 2006; Uttamchandani et al., 2006). Then, the entire sequence of the target protein is sequentially cut to equal lengths, and acquired peptides were administrated in the slide above. The peptides that show higher affinity to the receptor are chosen, and finally sequences are analyzed for further application. Owing to the outstanding advantages of miniaturization and parallelization, microarray chip technology has been widely used in peptide screening (Deng et al., 2020), antibody detection (Al‐Majdoub et al., 2013), and vaccine preparation (Gaseitsiwe et al., 2008). It should be noted that systematically screened sequences were usually short, mainly 8–15 amino acids, with partial simulation on domain structures of original protein. Improved mimic efficiency of biological function with moderate sequence is required in further motif studies before clinical application.

### 2.4 Microstructure simulation of functional domains

Microstructure simulation based on analyzing the microstructure of the functional domain, and adjusting the atomic arrangement and bonding angle to simulating the spatial structure. For example, Bhatnagar directly analyzed the structure of TGF-β, and proposed that the β-turn structure is of vital significance for its bioactivity. Then, a series of short peptides containing six or seven amino acids was developed, termed as cytomodulin (CM) family (Bhatnagar et al., 2003). Zhang *et al.* combined CM10 (LIANAK) with functional nanofibrous hollow microspheres (FNF-HMS), and implated subcutaneously in the backs of mice. Finally, ectopic cartilages were realized (Zhang et al., 2015a). However, the structure of functional domain is not fixed on different conditions, so as to realize the expose and block of bioactivity site. Simple structural simulation may not simultaneously satisfy the microscopic requirement in activation and inactivation of motifs.

## 3 Functional motifs functions for cartilage tissue engineering

The *in vivo* and *in vitro* studies about growth factor-relative functional motifs are summarized as follows (Table 1).

**TABLE 1:** Functional motifs applications for cartilage tissue engineering.

Growth factor	Function	Motif sequence	Selection mechanism	Carrier	Modification	Cell/Species	Results	Reference
TGF-β	Promote differentiation of MSCs into chondrocytes Blumenfeld et al. (1997)	ANVAENA (CM-1)	Microstructure simulation	—	—	Rat	CM1 improved the thickness of neotissue and collagen secretion in mouse wound model	El-Sakka et al. (1997)
	Improve cartilage matrix synthesis, such as collagen typeⅡand proteoglycans Garbuzenko et al. (2009)			—	—	hMSCs	CM1 improved the GAGs production, independent of dosage	Renner and Liu (2013)
	Reduce the activity of cytokines related to cartilage injury, such as interleukin-1 Sellers et al. (1997)	LIAEAK (CM-2)	Microstructure simulation	—	—	hMSCs	CM2 improved the GAGs production, independent of dosage	Renner and Liu (2013)
				CX-HA	Covalent bond	hPLSCs	Chemically crosslinked CM2 showed stabler release and better GAGs deposition, compared with physically mixed into HA hydrogel	Park et al. (2019)
		LIANAK (CM-10)	Microstructure simulation	FNF-HMS	Covalent bond	BMSCs	Safranin O, Von kossa and immunohistochemical stains showed better deposition of GAGs and collagen, with little calcification	Zhang et al. (2015a)
						Rat		
				—	—	Rat	CM10 induced early epithelialization and vascularization of skin wound, so as to strengthen the collagen deposition and tissue reconstruction	Basu et al. (2009)
BMHP	Recruitment of MSCs to cartilage defect areas Liu et al. (2013)	PFSSTKT	Highly repetitive conserved sequences	—	—	MNSCs	PFSSTKT recruited NSCs to local tissue, and the cell behaviors were stable, compared with Matrigel	Gelain et al. (2006)
				SAP hydrogel	Covalent bond	BMSC	RAD/PFS hydrogel did favor to the adhesion of rabbit BMSCs	Lu et al. (2018)
						Rabbit	Better reconstruction of articular cartilage was found after implanting RAD/PFS/ACM composite scaffold into rabbit knee cartilage defect	
		SKPPGTSS	Highly repetitive conserved sequences	—	—	MNSCs	SKPPGTSS recruited NSCs to local tissue, and the cell behaviors were stable, compared with Matrigel	Gelain et al. (2006)
				HX	Covalent bond	BMSC	BMSCs stayed healthy on different scaffolds	Sun et al. (2018)
						Rabbit	RAD/SKP/PFS group showed ideal neocartilage at rabbit knee cartilage defect area	
BMP	Drive the development of cartilage	KIPKASSVPTELSAISTYL	Phage Display technology	—	—	hMSCs	BMP-mimetic peptide significantly strengthened the secretion of GAGs in hMSCs	Renner and Liu (2013)
	Induce the differentiation of mesenchymal precursor cell into chondrocytes Yang et al. (2011)			—	—	hMSCs	BMP-mimetic peptide did favor for cartilage matrix deposition	Renner et al. (2012)
	Induce BMSCs to generate cartilage matrix both *in vitro* and *in vivo* Raducanu et al. (2009)						BMP-mimetic peptide reduced the secretion of collagen type X and the ALP activity of hMSCs	
		SYED (CK2.1)	Phage Display technology	—	—	Rat	CK2.1 increased the regeneration of cartilage but decreased the expression of collagen type X and osteocalcin	Akkiraju et al. (2017a)
				HGP	Covalent bond	Rat	CK2.1-HGP improved the cartilage restoration in mice but showed no evidence of hypertrophy, and lower deposition of collagen type X	Akkiraju et al. (2017b)
N-Cadherin	Mediate the aggregation and condensation of progenitor cells and MSCs Tavella et al. (1994)	HAV	Highly repetitive conserved sequences	MeHA hydrogel	Covalent bond	hMSCs	The productions of GAGs and collagen in HVA group were increased than other groups *in vitro* and *in vivo*	Bian et al. (2013)
						Rat		
				E-PA	Covalent bond	hMSCs	Cells adhered the HAV/E-PA network well	Eren Cimenci et al. (2019)
							Cells cultured on the HAV/E-PA scaffold secreted more GAGs, and showed higher expression of chondrogenic markers	
				KLD hydrogel	Covalent bond	hMSCs	With stimulation of HAVDI, the secretion of GAGs and gene expression of chondrogenesis were upgraded	Li et al. (2017a)
							The subcellular localization changed	
				—	—	hMSCs	HAV strengthened the expressions of early chondrogenic markers, depending on the dosage strongly	Kwon et al. (2018)
Integrin	Promote the adhesion between cells and ECM Place et al. (2009)	RGD	Highly repetitive conserved sequences	PEG hydrogel	Covalent bond	hPDC	Supplemented by RGD, cells survived and proliferated better	Kudva et al. (2018)
	Participate in the mechanical signal transduction pathway of chondrocytes Hajos et al. (2008)						The upregulation of cell spreading and downregulation of cell circularity confirmed the satisfying cell adhesion	
				Au-NPs	Covalent bond	hMSCs	Au-RGD1400 stimulation exhibited higher deposition of GAGs	Li et al. (2017b)
				PEG hydrogel	Covalent bond	Chondrocytes	RGD sequence was chemically crosslinked with PEG, resulting in more secretion of GAGs. A trend of hypertrophy in chondrocytes was found after stimulation of peptide RGD.	Zhang et al. (2015b)
				PEG hydrogel	Covalent bond	Chondrocytes	The risk of chondrocyte dedifferentiation tended to decrease when the microscopic distance were over 70nm, indicating more beneficial to maintain the normal phenotype of chondrocytes	Li et al. (2015)
				PEG hydrogel	Non-covalent bond	Chondrocytes	Without dynamic load, RGD had a negative effect on the phenotype of chondrocytes. Under dynamic compression, the expression of chondrogenic genes increased with the increase of RGD concentration	Villanueva et al. (2009)
		GRGDY	Highly repetitive conserved sequences	Calcium alginate hydrogel	Covalent bond	Chondrocytes	Formation of ectopic cartilage on the back of rats	Alsberg et al. (2002)
						Rat		
		CPENFFGGRGDSG	Highly repetitive conserved sequences	PEG hydrogel	Covalent bond	hMSCs	Enzymatically cleaved CPENFFGRGDSG showed limited long-term influence on cell viability. With stimulation of CPENFFGRGDSG, the secretion of GAGs was significantly improved	Salinas and Anseth (2008)
IGF	Induce the proliferation and chondrogenic differentiation of MSCs Trippel, (1995)	GRVDWLQRNANFYDWFVAELG	Phage Display technology	—	—	hMSCs	Insulin-derived peptide of 0.1 μM improved the deposition of GAGs, with the presence of TGF-β3	Renner and Liu (2013)
PTH	Induce MSCs to differentiate into chondrocytes, but counteracting hypertrophic differentiation, so as to maintain the phenotype of chondrocyte Jiang et al. (2008)	PtHrP	Highly repetitive conserved sequences	—	—	BMSC	The content ratio of collagen type II to collagen type I was significantly improved by PTHrP	Kafienah et al. (2007)
		(1–34)					The expression of collagen type X was significantly downregulated by PTHrP	
				—	—	MSCs, hAC Rat	PTHrP inhibited the ALP activity and gene expression of Indian hedgehog and collagen type X	Fischer et al. (2014)
				—	—	MSCs	The deposition of proteoglycan and collagen type II was promoted, and decreased expression trend of collagen type X was found	Rajagopal et al. (2021)
				—	—	MSCs	PtHrP supplementation from day 4 significantly increased the expression of chondrogenic markers compared with day 14	Zhang et al. (2013)
				—	—	BMSC	PTHrP improved the chondrogenic matrix deposition of proteoglycan and collagen type II.	Kim et al. (2008)
						ADSC	The markers of endochondral osteogenesis were inhibited	
				—	—	NC, MSC	Implanted cell pellets that treated with PTHrP showed improved Safranin-O staining and anti-collagen type I/II IF staining results after 3 weeks	Johnstone et al. (1998)
						Rat	Weakly positive stains of Alizarin Red S and anti-collagen type X/CD31 IF staining were found	
		PTHrP (1–40)	Highly repetitive conserved sequences	—	—	Rabbit	The time window between 4 and 6 weeks for PTHrP injection benefited the rat knee cartilage repair better	Anderson-Baron et al. (2021)

### 3.1 TGF-β-related motifs

**FIGURE 2:**
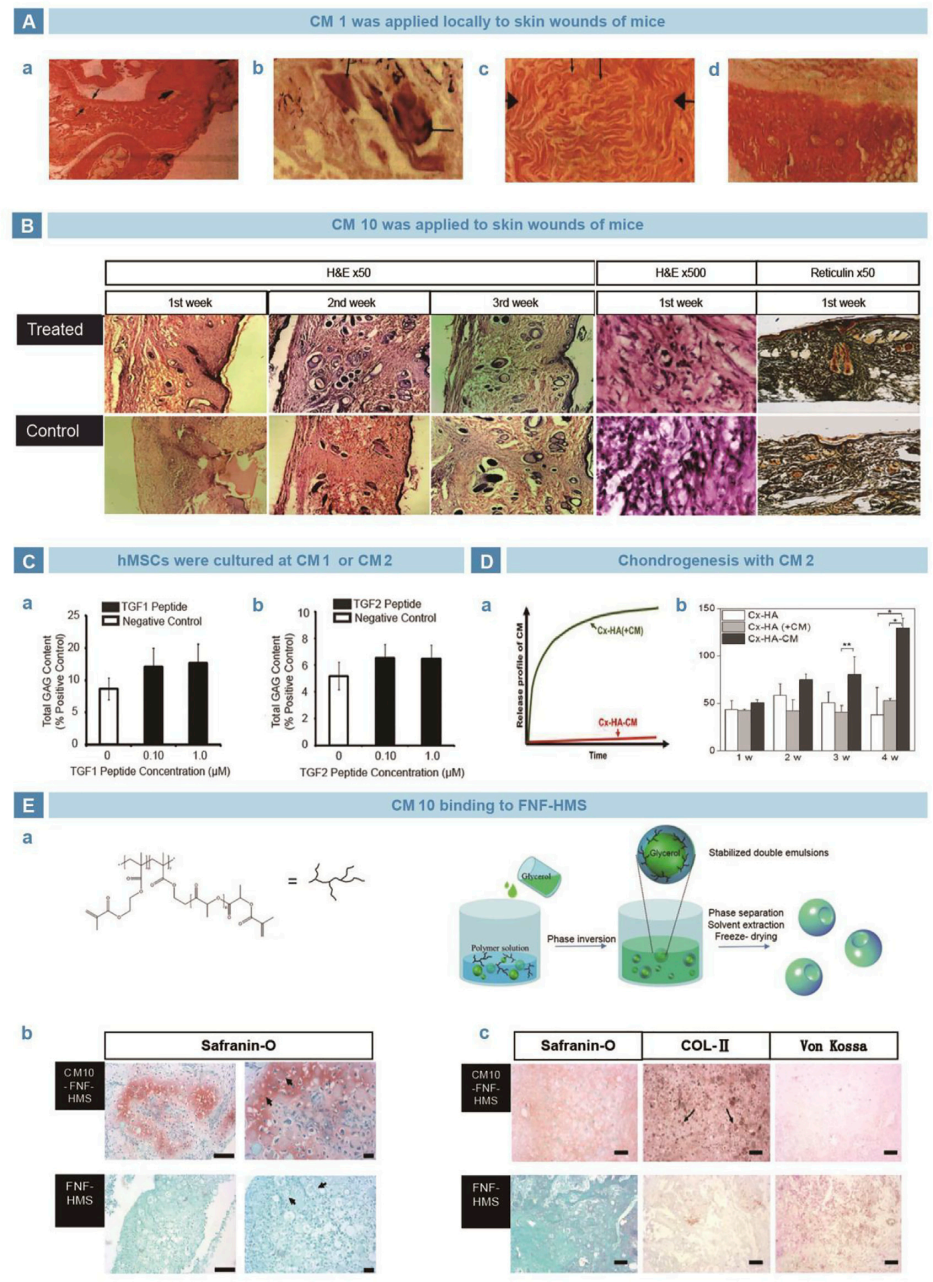
TGF-β mimetic peptide CMs in cartilage regeneration. **(A)**. CM1 was used in mouse wound model and improved the repair efficiency of neotissue thickness **(A)**, calcification **(B)**, collagen secretion **(C)**, compared with control group **(D)** (El-Sakka et al., 1997). **(B)**. CM10 induced early epithelialization and vascularization of skin wound, so as to strengthen the collagen deposition and tissue reconstruction (Basu et al., 2009). **(C)**. Administration of CM1 and CM2 improved the GAGs production, independent of dosage (Renner and Liu, 2013). **(D)**. Compared with physically mixed into HA hydrogel, chemically crosslinked CM2 showed stabler release curve **(A)** and better GAGs deposition **(B)** (Park et al., 2019). **(E)**. CM10 was loaded on nanofiber hollow microspheres prepared by emulsification and phase separation **(A)**. Safranin O staining exhibited significantly higher GAGs secretion *in vitro* **(B)**. After implanted subcutaneously in rats, the Safranin O, Von kossa and immunohistochemical stains showed better deposition of GAGs and collagen, with little calcification **(C)** (Zhang et al., 2015a).

TGF-β is a family of proteins that regulates key cellular processes involved in early embryonic development (Peng et al., 2022), cell growth (Frangogiannis, 2022), differentiation (Moreau et al., 2022), motility, and apoptosis (Weiss and Attisano, 2013). As known to all, TGF-β is vital in maintaining articular cartilage normality and joint integration. Bhatnagar selected a series of short peptides containing six or seven amino acids from the structural characteristics of TGF-β, named as the CM family (Bhatnagar et al., 2003), and main members include CM1 (ANVAENA), CM2 (LIAEAK), and CM10 (LIANAK). El-Sakka (El-Sakka et al., 1997) and Basu (Basu et al., 2009)applied CM1 and CM10 locally in mouse skin wound model, and found improvement in collagen I expression and wound strength (Figure 2A, B). Renner *et al.* cultured human mesenchymal stem cells (hMSCs) with free CM1 and CM2, but no significant difference was found in glycosaminoglycan (GAG) production, compared with negative controls (Figure 2C) (Renner and Liu, 2013). In contrast, Park *et al.* added CM2 into medium to culture human periodontal ligament stem cells (hPLSCs) and found increased expression of SOX9, ACAN, and COL2A1. Moreover, compared with the non-covalent binding mode, the covalently combined CM2 with Cx-HA exerted longer influence on GAG deposition (Figure 2D) (Park et al., 2019). Similarly, Zhang *et al.* covalently grafted CM10 onto FNF-HMS and found that the functionalized scaffold strengthened chondrogenic differentiation *in vitro*. Then the scaffold were injected subcutaneously into mice, which showed ectopic cartilage formation (Figure 2E) (Zhang et al., 2015a).

### 3.2 BMPH-related functional motifs

Hyaline cartilage locates at the end of the long bone, lacks blood supply and reserve cells. Its lubrication and feeding relies on articular fluid at the joint capsule. Similarly, the poor self-repair ability of articular cartilage defects is poor for that the chemokines are usually too insufficient in the defect area to guide the accumulation of stem cells (Chen et al., 2021). For example, BMHP is able to enter bone marrow and bind to BMSCs *in vivo*, which does favor to induce MSCs to migrate, proliferate, differentiate and synthesize matrix to repair cartilage defect (Liu et al., 2013).

**FIGURE 3:**
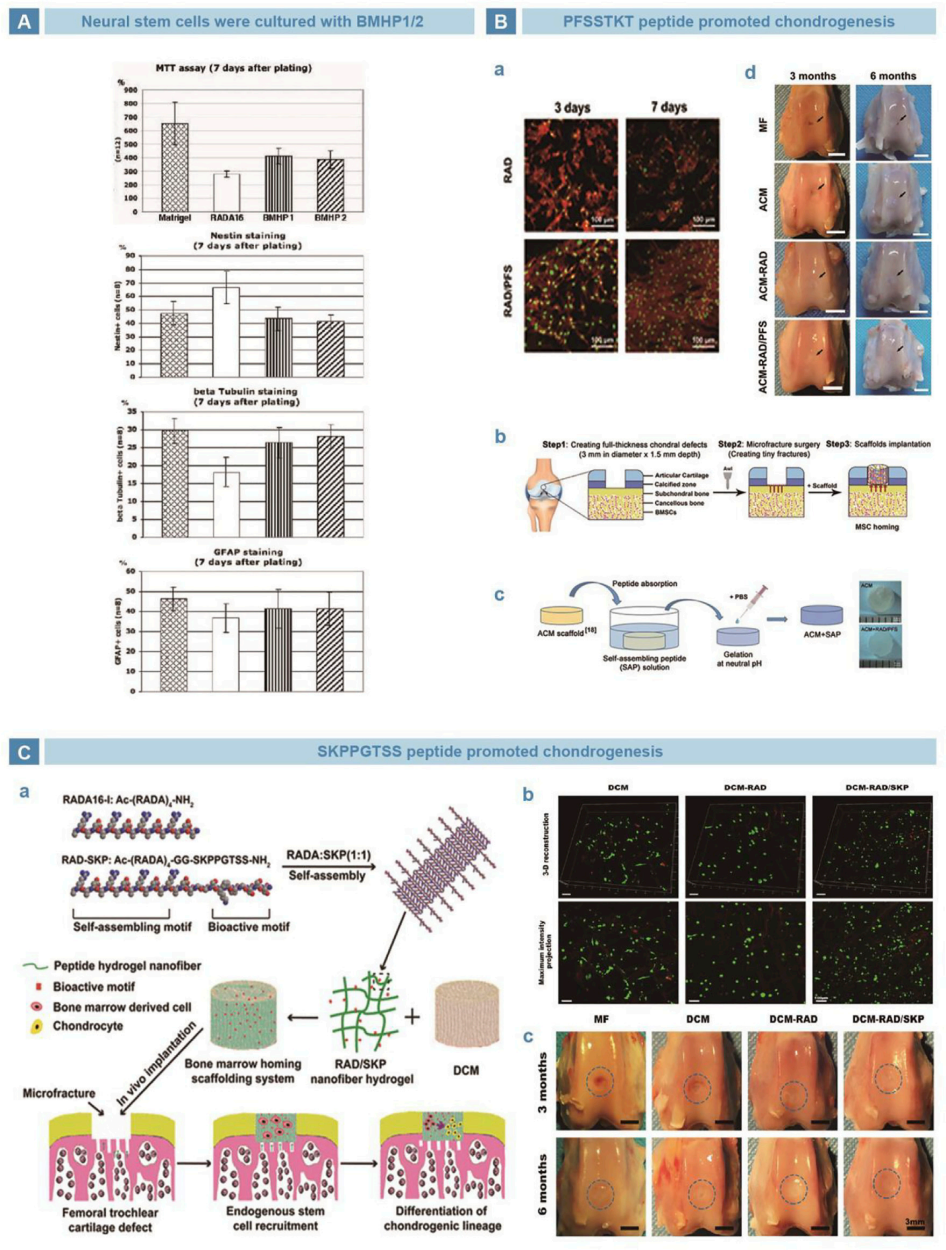
BMHP-mimetic peptides, PFSSTKT and SKPPGTSS in tissue engineering. **(A)**. BMHP1 (PFSSTKT) and BMHP2 (SKPPGTSS) recruited NSCs to local tissue, and the cell behaviors were stable, compared with Matrixgel (Gelain et al., 2006). **(B)**. PFSSTKT in cartilage tissue engineering. RAD/PFS hydrogel did favor to the adhesion of rabbit BMSCs **(A)**. RAD/PFS was merged in acellularized cartilage matrix scaffold to acquire functional composite scaffold, then implanted into full-depth rabbit knee cartilage defect **(B)**. PFSSTKT was conjugated with peptide RADA-16 I to prepare functional self-assembling peptide hydrogel RAD/PFS **(C)**. Better reconstruction of articular cartilage was found after implanting RAD/PFS/ACM composite scaffold into rabbit knee cartilage defect **(D)** (Lu et al., 2018). **(C)**. SKPPGTSS in cartilage tissue engineering. Conjugation of RAD and SKPPGTSS, and fabrication of composite scaffold of RAD/SKP/DCM **(A)**. BMSCs stayed healthy on different scaffolds **(B)**. RAD/SKP/PFS group showed ideal neocartilage at rabbit knee cartilage defect area **(C)** (Sun et al., 2018).

Nowakowski et al. (2004) screened a series of peptides with strong affinity for stem cells, and found that the amino acids K, *p*, F, S, and T are crucial for biological functions Subsequently, Gelain *et al.* verified the bioactivities of BMHP1 (PFSSTKT) and BMHP2 (SKPPGTSS), as mimetic peptides of BMHP that rich in amino acids K, *p*, F, S, and T. Stem cell recruitment was realized with the two peptides as well as an improved trend of cell differentiation (Figure 3A) (Gelain et al., 2006). Lu *et al.* combined the PFSSTKT short peptide with RAD to produce a functionalized SAP hydrogel, which stimulated MSC proliferation, attachment, and chondrogenic differentiation in rabbit model. Acellularized cartilage matrix (ACM) scaffold was combined with SAP hydrogel to form ACM + RAD/PFS and implanted into full-thickness articular cartilage defect area, and it was found that the cartilage defect was completely covered by chondroid tissue (Figure 3B) (Cao et al., 2015; Lu et al., 2018). Sun *et al.* introduced the SKPPGTSS short peptide and RAD into a three-dimensional (3D) porous decellularized porcine articular cartilage matrix (DCM) scaffold to form a bone marrow-specific homing scaffold system, DCM-RAD/SKP. Experiments *in vitro* showed that rabbit bone marrow stem cells migrated to DCM-RAD/SKP scaffold, and the cell/scaffold system were implanted into rabbit knee cartilage defect model. Computed Tomography (CT) results showed that the cartilage defect area was filled with uniform regenerated tissue, highly similar with surrounding normal cartilage, and successfully reconstructed the subchondral bone (Figure 3C) (Sun et al., 2018). According to studies above, the peptides PFSSTKT and SKPPFTSS successfully recruited stem to specific location, in spite of original tissue. However, PFSSTKT and SKPPFTSS exerted limited effect on differentiation, indicating that differentiation depends on other conditions, such as environment.

### 3.3 BMP-related functional motifs

**FIGURE 4:**
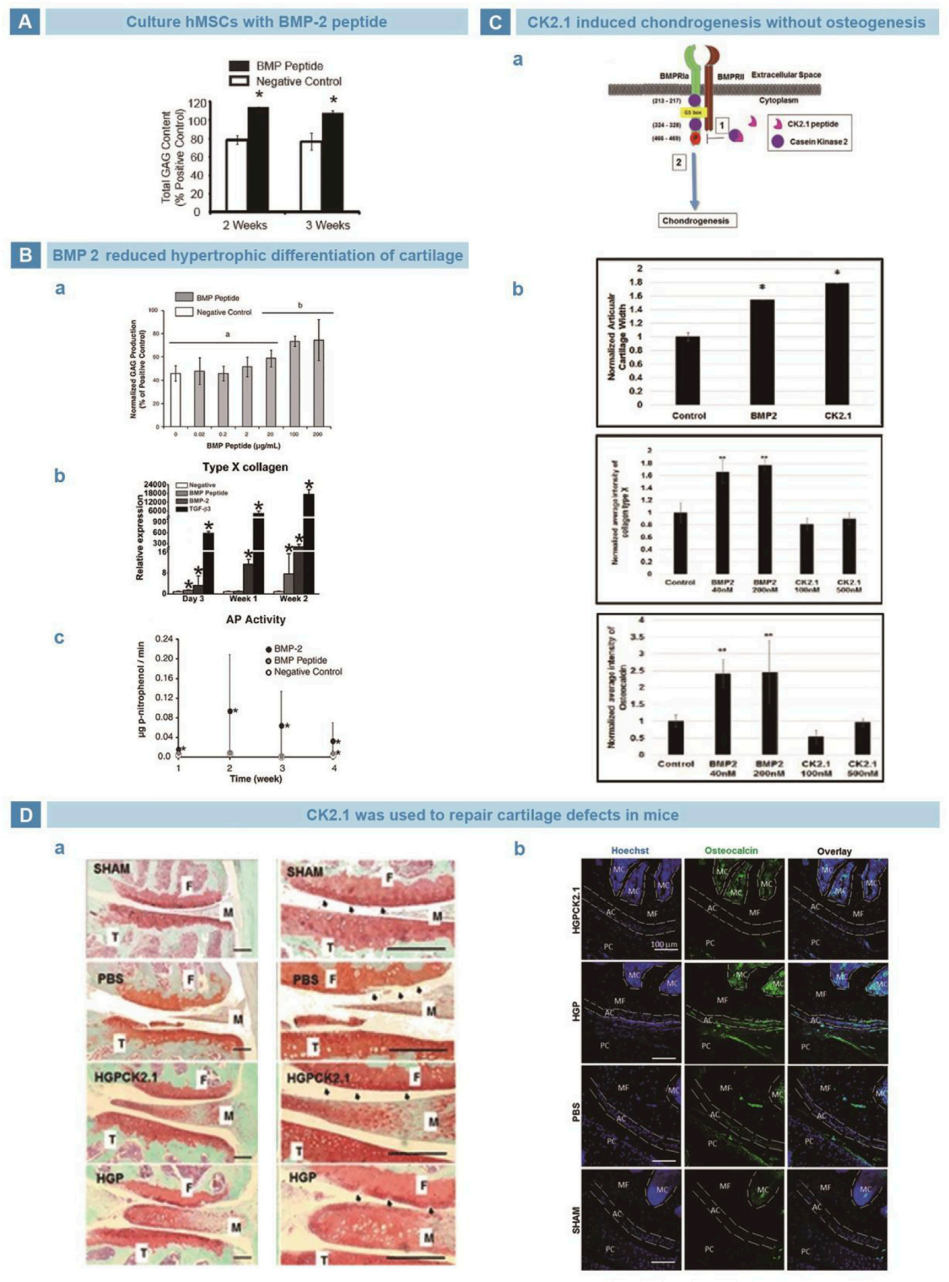
BMP-related peptides in cartilage regeneration. **(A)**. BMP-mimetic peptide significantly strengthened the secretion of GAGs in hMSCs (Renner and Liu, 2013). **(B)**. BMP peptide maintained the cartilage structure. BMP peptide did favor for cartilage matrix deposition **(A)**. BMP peptide reduced the secretion of collagen type X **(B)**. BMP peptide decreased the ALP activity of hMSCs **(C)** (Renner et al., 2012). **(C).** Mimetic peptide of BMP receptor type Iα, CK2.1, suppressed hypertrophy and ossification risk of hMSCs. Schematic illustration of CK2.1 **(A)**. CK2.1 increased the regeneration of cartilage but decreased the expression of collagen type X and osteocalcin **(B)** (Akkiraju et al., 2017a). **(D)**. CK2.1-HGP improved the cartilage restoration in mice but showed no evidence of hypertrophy **(A)**, and lower deposition of collagen type X **(B)** (Akkiraju et al., 2017b).

BMPs are members of TGF-β family, and play an important role in regulating cell behavior and tissue regeneration (Zhou et al., 2022). BMPs participate in the differentiation of MSCs into bone, cartilage, ligaments, tendons, and nerves (An et al., 2010). Saito *et al.* Found that KIPKASSVPTELSAISTLYL, a 20-amino acid sequence from residues 73–92 of BMP2, may be one of the receptor binding sites to induce calcification of fibroblasts (Saito et al., 2003). Renner *et al.* used KIPKASSVPTELSAISTLYL to culture hMSCs, and found the GAG production was higher after 2 weeks (Figure 4A) (Renner and Liu, 2013). More importantly, Kim *et al.* found that compared with BMP2 group, cells treated with KIPKASSVPTELSAISTLYL exhibited almost no increase in hypertrophy markers but significantly increased secretion of cartilage matrix (Figure 4B) (Renner et al., 2012). In addition, Akkiraju *et al.* designed a novel mimic peptide CK2.1(Syed) from BMP receptor Ia (BMPRIa) and injected it into the tail veins of mice. CK2.1 promoted chondrogenesis without inducing chondrocyte hypertrophy, and the effect was better than that of BMP (Figure 4C) (Akkiraju et al., 2017a). Subsequently, Akkiraju injected CK2.1-loaded hydrogel particles (HGP) into articular cartilage defects of mice, and found that defects were filled with typical neocartilage of less hypertrophy (Figure 4D) (Akkiraju et al., 2017b).

### 3.4 N-cadherin-related functional motifs

N-cadherin is a calcium ion-dependent adhesion glycoprotein and function in maintaining cell structure and motility (Tepass et al., 2000). In chondrogenesis, N-cadherin mediates the aggregation and condensation of mesenchymal cells, like chondrogenic progenitor cells (Tavella et al., 1994; Richardson et al., 2007). Gao suggested that N-cadherin-mediated cell-cell interactions were of great significance in mesenchymal cell densification and chondrogenesis (Gao et al., 2010).

**FIGURE 5:**
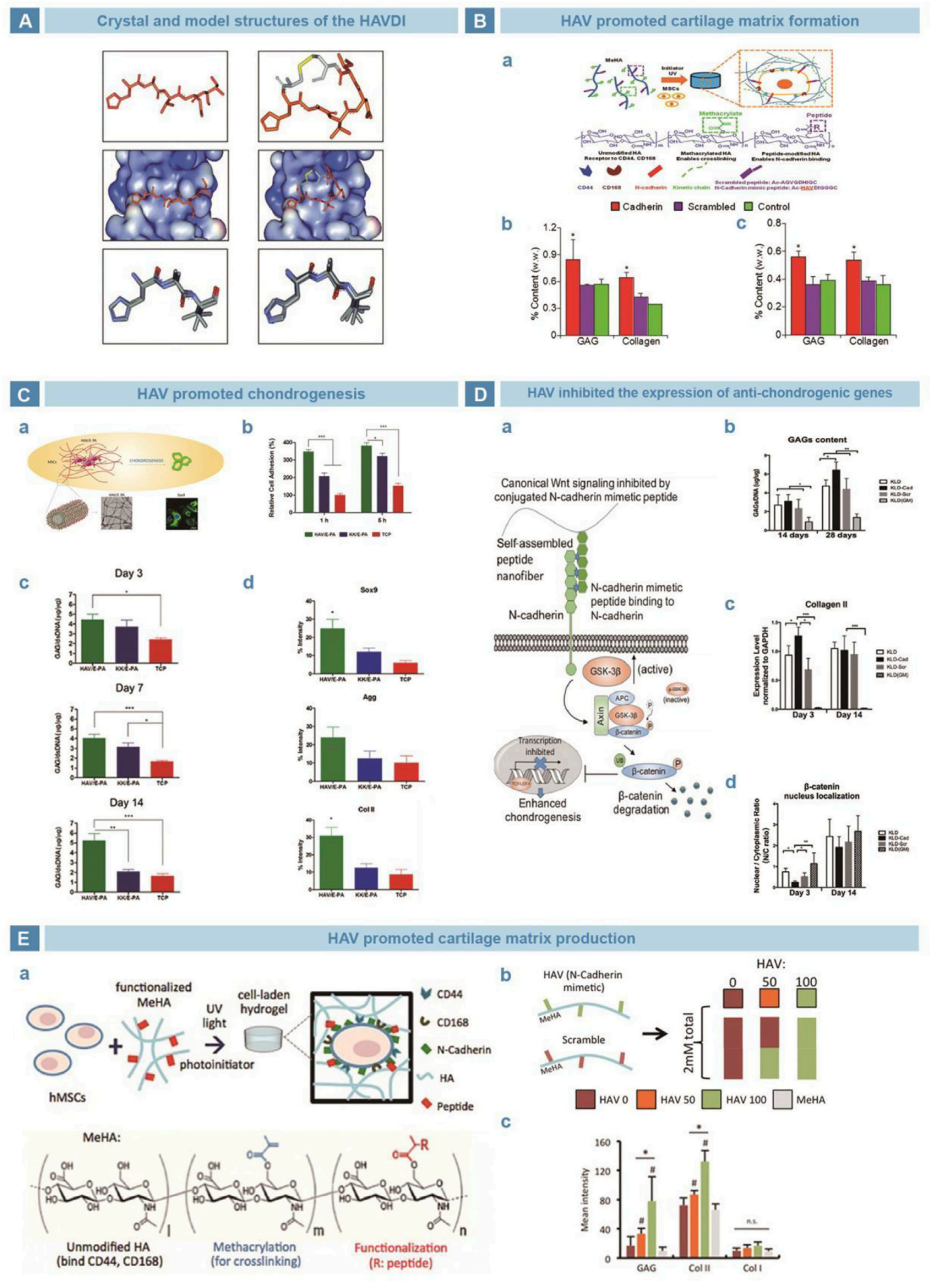
N-cadherin mimetic peptide HAV in cartilage engineering. **(A)**. Crystal structure of HAVDI (Williams et al., 2000). **(B)**. Tripeptide HAV in chondrogenesis. HAV was grafted onto MeHA hydrogel **(A)**, and the productions of GAGs and collagen were evaluated *in vitro* **(B)** and *in vivo* **(C)** (Bian et al., 2013). **(C)**. HAV was grafted with E-PA to get nanofiber network **(A)**. Cells adhered the HAV/E-PA network well **(B)**. Cells cultured on the HAV/E-PA scaffold secreted more GAGs **(C)**, and showed higher expression of chondrogenesis (Eren Cimenci et al., 2019). **(D)**. Self-assembling peptide KLD-HAVDI mimic the functional domain of N-cadherin **(A)**. With stimulation of HAVDI, the secretion of GAGs and genetic expression of chondrogenesis were upgraded **(B–C)**. Meanwhile, the subcellular localization changed **(D)** (Li et al., 2017a). **(E).** HAV was grafted to MeHA for crosslinking **(A)**, and hydrogels of different proportion were prepared **(B)**. HAV strengthened the expressions of early chondrogenic markers, depending on the dosage strongly **(C)** (Kwon et al., 2018).

Williams *et al.* found that N-cadherin has an evolutionarily conserved sequence HAV (Williams et al., 2000), which provides a homophile cell adhesion recognition site and mediates cell-cell adhesion (Blaschuk et al., 1990). Relative studies clarified that inhibition of the HAV peptide weakened cell-cell adhesion (Figure 5A) (Williams et al., 2000). Williams *et al.* performed a series of amino acid modifications on the basic HAV sequence and synthesized peptides showed similar affinity with N-cadherin (Williams et al., 2001). Bian incorporated HAV into a MeHA hydrogel to promote the early expression of chondrogenic genes and latter cartilage-specific matrix production (Figure 5B) (Bian et al., 2013). Cagla *et al.* combined HAV and the amphiphilic peptide nanofiber system E-PA, and realized promoted mesenchymal cell cohesion and cartilage formation (Figure 5C) (Eren Cimenci et al., 2019). Li chemically grafted HAVDI with a KLD12 self-assembled peptide hydrogel and found that the nuclear translocation of β-catenin and the synthesis of type X collagen at the early stage of chondrogenesis. By the downregulation of the WNT/β-catenin pathway, the expression of chondrogenic genes was improved and cartilage matrix was preserved (Figure 5D) (Li et al., 2017a). Kwon *et al.* suggested that the HAV peptide enhanced the early expression of chondrogenic markers and promoted the long-term deposition of cartilage matrix in a strongly dose-dependent manner (Figure 5E) (Kwon et al., 2018).

### 3.5 Integrin-related functional motifs

**FIGURE 6:**
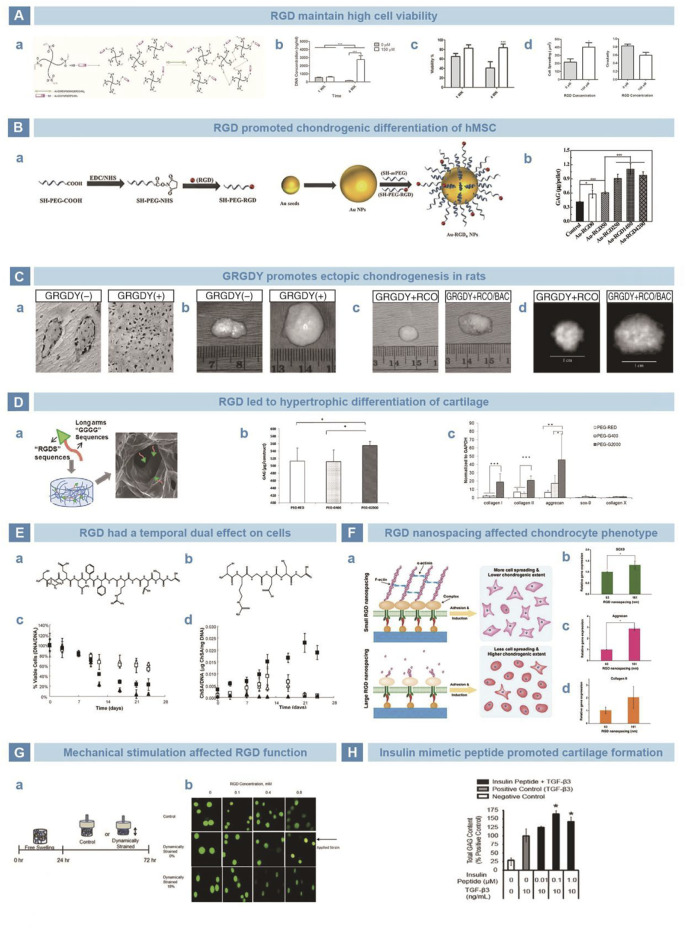
Peptides mimicking integrin and insulin in cartilage restoration. **(A)**. RGD sequence functioned the PEG-VS was crosslinked *via* di-thiol crosslinker **(A)**. Supplemented by RGD, cells survived and proliferated better **(B–C)**. Besides, the upregulation of cell spreading and downregulation of cell circularity confirmed the satisfying cell adhesion **(D)** (Kudva et al., 2018). **(B)**. Peptide RGD was conjugated onto Au-nanoparticals **(A)**. Au-RGD1400 stimulation exhibited higher deposition of GAGs **(B)** (Li et al., 2017b). **(C)**. Peptide G-RGD-Y stimulated chondrocytes turned out typical collagen distribution by Masson stain **(A)**. The bovine articular chondrocytes and calvarial osteoblasts were mixed and loaded into RGD-modified alginate scaffold, which turned into neocartilage of larger size **(B)**, mass **(C)** and high-density image under X-ray **(D)** (Alsberg et al., 2002). **(D)**. RGD sequence was chemically crosslinked with PEG **(A)**, resulting in more secretion of GAGs **(B)**. However, a trend of hypertrophy in chondrocytes was found after stimulation of peptide RGD **(C)** (Zhang et al., 2015b). **(E)**. Chemical formulas of enzymatically cleavable peptide CPENFFGRGDSG **(A)** and uncleavable peptide CRGDSG **(B)**. Enzymatically cleaved CPENFFGRGDSG showed limited long-term influence on cell viability **(C)**. With stimulation of CPENFFGRGDSG, the secretion of GAGs was significantly improved **(D)** (Salinas and Anseth, 2008). **(F)**. RGD was grafted on PEG hydrogel to stimulate MSCs **(A)**. Expression of chondrogenic genes on different nanospacings after 10 days **(B–D)** (Li et al., 2015). **(G)**. Dynamic mechanical loading was exerted on MSCs after 24-h cultivation **(A)**. RGD alleviated the trend of morphology change after straining **(B)** (Villanueva et al., 2009). **(H)**. Insulin-derived peptide of 0.1 μM improved the deposition of GAGs, with the presence of TGF-β3 (Renner and Liu, 2013).

Integrin is an adhesion protein on the membrane that can transmit signals by interacting with extracellular matrix (ECM), so as to regulate key cellular processes, such as cell differentiation, proliferation and migration (LaFlamme et al., 2018). Interestingly, integrin can improve the adhesion between cells and ECM, and allow cells to adapt to the surrounding environment better (Place et al., 2009). RGD sequence has been found in multiple ECM proteins that promote cell adhesion, such as fibronectin (Underwood et al., 1995), laminin (Wang et al., 2021), tenascin (Comisar et al., 2007), and thrombospondin (Benoit and Anseth, 2005). RGD binds integrins to anchor cells on the surface of matrix and enhance cell migration, adhesion, extension, and proliferation. Patterson *et al.* grafted RGD to polyethylene glycol (PEG) hydrogels to culture human periosteum-derived cells (hPDCs). The cells maintained high viability and promoted the expression of chondrogenic genes and synthesis of GAGs (Figure 6A) (Kudva et al., 2018). Li *et al.* conjugated RGD to gold nanoparticles (Au-NPs) and improved differentiation of hMSCs into chondrocytes (Figure 6B) (Li et al., 2017b). Alsberg *et al.* injected mixture of calcium alginate hydrogel-GRGDY-chondrocytes subcutaneously at the backs of rats, and found typical ectopic chondrogenesis (Figure 6C) (Alsberg et al., 2002). However, Zhang *et al.* suggested that RGD peptides promote chondrocyte proliferation and differentiation, but simultaneously lead to chondrocyte hypertrophy and differentiation (Figure 6D) (Zhang et al., 2015b). Salinas *et al.* believed that the influence of RGD on cells based on time. When the cleavable CPENFFGGRGDSG system was added to the PEG hydrogel loaded with hMSCs, early chondrogenesis could be induced. The removal of RGD reduced the risk of inhibited chondrogenesis (Figure 6E) (Salinas and Anseth, 2008). Li *et al.* cultured chondrocytes in PEG hydrogel loaded with RGD. The chondrocyte dedifferentiation was inhibited when the microscopic distance was over 70 nm. Then, 70 nm was consider the most beneficial distance of RGD stimulation to maintain the phenotype of chondrocytes (Figure 6F) (Li et al., 2015). Mechanical stimulation was also thought of as one factor that affects RGD function, according to the role of integrin in the mechanical signal transduction pathway of chondrocytes. Studies by Villanueva showed that RGD has a negative effect on the phenotype of chondrocytes without a dynamic load. However, the phenotype of chondrocytes could be maintained after dynamic compression (Figure 6G) (Villanueva et al., 2009).

### 3.6 IGF-related functional motifs

Both insulin and insulin-like growth factor 1 (IGF1) play key roles in chondrogenesis. Insulin, an important component in almost all chondrogenic supplements (Puetzer et al., 2010), was reported to be significant in chondrocyte redifferentiation and could independently induce cartilage matrix synthesis of chondrogenic cell line ATDC5 (Shukunami et al., 1996). IGF1 is an important growth factor in chondrogenesis and the regulator of cartilage homeostasis. IGF1 promotes expression of chondrogenic gene, synthesis of collagen type II and proteoglycans, cell proliferation, and inhibits matrix decomposition mediated by osteoclasts (Trippel, 1995). IGF1 was found to interact with insulin receptors. Therefore, insulin-related peptides may also play a role similar to that of insulin and IGF1 for cross-reactivity (Phornphutkul et al., 2006). Renner *et al.* designed an insulin-mimetic peptide, GRVDWLQRNANFYDWFVAELG (GRV), which exhibited a high affinity for the insulin receptor. Then, the peptide GRV at different dosage of 0.01 µM, 0.1 µM, and 1 µM were used to culture hMSC. The results showed that differentiated chondrocytes, originating from hMSCs containing peptide GRV and TGF-β3, secreted more GAG than the control group of TGF-β3. It is believed that, with the presence of insulin and TGF-β3, the insulin functional motif GRV promoted chondrogenic differentiation and cartilage matrix deposition (Figure 6H) (Renner and Liu, 2013).

### 3.7 Parathyroid hormone (PTH) functional motifs

**FIGURE 7:**
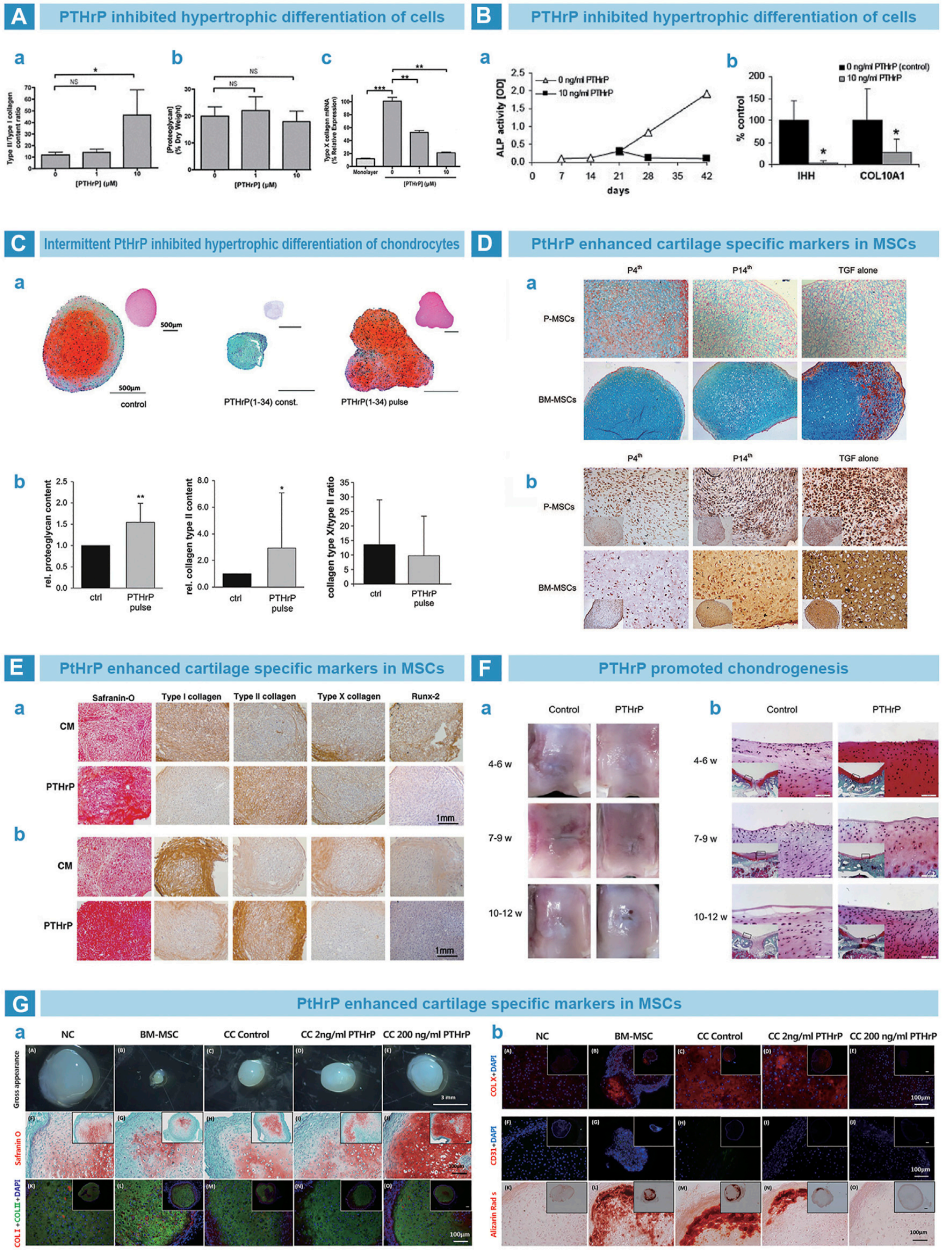
PTH-related peptide (PTHrP) in osteoarthritis. **(A)**. PTHrP prevented the hypertrophy of chondrocytes. Collagen quantification results that the content ratio of collagen type II to collagen type I was significantly improved by PTHrP **(A)**, while the proteoglycan exhibited no difference by DMMB assay **(B)**. However, the expression of collagen type X was significantly downregulated by PTHrP, as qPCR results (Kafienah et al., 2007). **(B)**. PTHrP inhibited the endochondral indexes of ALP activity and mRNA expression of Indian hedgehog and collagen type X (Fischer et al., 2010). **(C)**. Pulsative stimulation of PTHrP was performed on chondrocytes **(A)**. The deposition of proteoglycan and collagen type II was promoted **(B)**, and decreased expression trend of collagen type X was found **(C)** (Fischer et al., 2014). **(D)**. Supplemented PTHrP from fourth day strengthened the cartilage matrix deposition of periosteal MSCs and bone marrow MSCs by Alcian blue **(A)**, but suppressed the endochondral osteogenesis, as immunohistochemical (IHC) staining of collagen type X **(B)** (Rajagopal et al., 2021). **(E)**. PTHrP improved the chondrogenic matrix deposition of proteoglycan and collagen type II by Safranin-O staining and IHC staining **(A)**. However, the markers of endochondral osteogenesis were inhibited, such as collagen type I, collagen type X and Runx-2 by IHC staining (Kim et al., 2008). **(F)**. The time window between 4 and 6 weeks for PTHrP injection benefited the rat knee cartilage repair best, as the result of gross morphology **(A)** and Safranin-O staining **(B)** (Zhang et al., 2013). **(G)**. Implanted cell pellets that treated with PTHrP showed improved Safranin-O staining and anti-collagen type I/II IF staining after 3 weeks **(A)**. Meanwhile, weakly positive stains of Alizarin Red S and anti-collagen type X/CD31 IF staining were found **(B)** (Anderson-Baron et al., 2021).

PTH is a single-chain polypeptide hormone synthesized and secreted by parathyroid cells (Suva and Friedman, 2022). PTH can regulate calcium and phosphorus metabolism at bone, kidneys and small intestine (Lombardi et al., 2020). The differentiation of MSCs into chondrocytes is usually accompanied with terminal hypertrophic differentiation (Fischer et al., 2010). PTH guides the differentiation of MSCs into chondrocytes (Chang et al., 2009) but counteracts hypertrophic differentiation (Jiang et al., 2008), so as to maintain the pehnotype of chondrocytes (Kim et al., 2008; Weiss et al., 2010). Suva *et al.* (Moseley et al., 1987) found that parathyroid hormone-associated protein (PTHrP) and PTH share similar sequence at residues 1–34, and exert similar effects by the same receptor, PTH1R (Weisser et al., 2002). Kafienah et al. (2007) *.* demonstrated that PTHrP can inhibit the hypertrophic differentiation of BMSC (Figure 7A) Fischer *et al.* cultured BMSCs with PTHrP in conditioned medium from human articular chondrocytes, and successfully reduced the expression of hypertrophy markers such as COL10A1 and alkaline phosphatase (Figure 7B) (Fischer et al., 2010). Meanwhile, Fischer *et al.* cultured MSCs in an environment of continuous or intermittent PtHrP(1–34) stimulation. The results showed that continuous stimulation inhibited chondrogenic differentiation of MSCs, whereas intermittent stimulation increased the deposition of cartilage matrix but inhibited hypertrophic differentiation (Figure 7C) (Fischer et al., 2014). In addition, the point of stimulation time on chondrogenesis was also studued. Rajagopal *et al.* supplemented PtHrP (1–34) into chondrogenic medium from day 4, and found that chondrogenic markers were significantly increased. At the same time, hypertrophy markers were significantly reduced compared to those treated with PtHrP from day 14 (Figure 7D) (Rajagopal et al., 2021). Kim et al. (2008) supplemented PTHrP (1–34) into chondrogenic medium for BMSCs and adipose tissue mesenchymal stem cells (ADSCs) from 14th day. The staining results indicated promoted chondrogenesis and inhibited hypertrophy *in vitro* (Figure 7E) (Kim et al., 2008). Zhang et al. (2013) injected PTHrP(1–40) in the articular cavity of rabbits at three time points after osteochondral injury. The results showed that, at 4–6 weeks group, articular cartilage exhibited better morphology and lower expression of hypertrophy markers than others (Figure 7F) (Zhang et al., 2013). Baron *et al.* cocultured human nasal septal chondrocytes with MSCs (NC/MSC) in a 1:3 ratio and added PTHrP. The results indicated that PTHrP inhibited the expression of hypertrophic markers in a dose-dependent manner and promoted secretion of cartilage matrix. PTHrP was found to eliminate cartilage calcification *in vivo* after the implantation of PTHrP-containing pellets into immunodeficient nude mice. In contrast, the control group exhibited obvious cartilage calcification (Figure 7G) (Anderson-Baron et al., 2021).

## 4 Carriers of existing functional motifs

**FIGURE 8:**
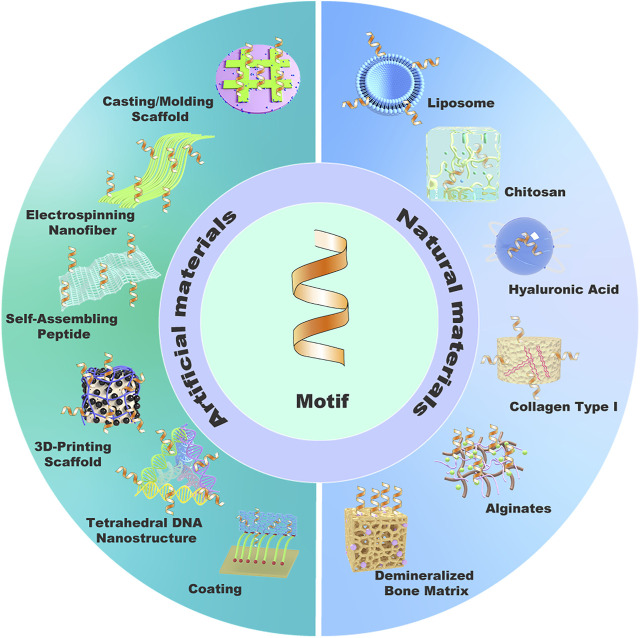
Carrier classifications based on material source.

Protein-mimetic peptides require suitable scaffold carriers to release in the recipient area, so as to achieve desired repair effect. Therefore, it is significantly meaningful to design safe, effective, and stable carriers for transporting functional motifs. Current scaffold carriers of motif in cartilage tissue engineering were as follows, including artificial materials and natural materials (Figure 8).

### 4.1 Natural materials

Natural materials, such as liposomes, collagen, and polysaccharide compounds, are popular for wide sources and low cost. In addition, they show high stability, safety, hydrophilicity, and other advantages. However, disadvantages also exist, such as low drug loading and poor adhesion to cells. Therefore, natural materials need modification or combination with other materials.

#### 4.1.1 Liposomes

Since liposomes were discovered by Bangham in the 1960s, their unique structure and properties exhibited excellent application prospects as carriers of oligonucleotides (Garbuzenko et al., 2009), polypeptides (Hajos et al., 2008), and proteins (Liu et al., 1993). As carriers, liposomes have many advantages. 1) Targeted effect. The structure of liposomes is similar to that of the vesicles (Rideau et al., 2018), which can directly enter cells by endocytosis of target cells. 2) Low immunogenicity, low-toxicity, and high-biocompatibility. 3) Broad drug loading. For their bimolecular lipid layer structure, liposomes can load lipid-soluble drugs between lipid membranes, amphiphilic drugs on phospholipids, and hydrophilic drugs in the aqueous phase. However, some disadvantages of liposomes should be noticed. Firstly, designing and applying the nano-delivery of liposomes require complicated technologies. Secondly, the cost of liposome formulations in industrialized production remains high. Finally, the stability and targeting, as well as potential toxicity of liposomal delivery systems are insufficient. Therefore, it is of great significance to solve the problems before wide application of liposome in clinical.

#### 4.1.2 Collagen

Collagen is a main component of connective tissue in mammals and structural component of bones (Halper and Kjaer, 2014).There are different types of collagen. Previous researches found collagen type I mainly in skin, tendon tissues and fibrous cartilage, collagen type II mainly in hyaluronan cartilage, collagen type X mainly in hydrophobic cartilage (Ricard-Blum, 2011). In clinical, collagen type I is the most popular collagen as carrier materials of peptide motifs. Collagen possesses multiple advantages (Gelse et al., 2003). 1) Sufficient sources to acquire and extract. 2) Low-immunogenicity, low-toxicity and good biocompatibility. 3) Long-term release behavior (Maeda et al., 2001). 4) Excellent structure plasticity (Wallace and Rosenblatt, 2003). 5) Various bioactivities, such as promotion of blood clotting. However, some disadvantages exist. 1) High cost of purification. 2) Rapid swelling and degradation due to hydrophilicity. 3) Poor mechanical properties. Therefore, the application of collagen as carriers requires more exploration.

#### 4.1.3 Demineralized bone matrix (DBM)

DBM based on removing bone mineral components but preserving natural protein components, calcium-based solids, inorganic phosphates and polysaccharides. In 1965, Urist found that DBM can induce osteogenesis (URIST and STRATES, 1970). Decalcification can expose and activate osteo-inductive proteins, enabling allogeneic bone to have an active osteo-inductive capacity for implanted bone resorption and new bone formation (Gloivacki, 1985). In addition, DBM has good biocompatibility, bioactivity, and biodegradability, and is easy to integrate into the surrounding bone and cartilage, so as to support the restoration of local tissue (Zhang et al., 2019). As a popular biomaterial, DBM still has a few difficulties. Firstly, the structure of DBM is too loose to fixed at the defect area. Secondly, the DBM of standard morphology usually could not fit the defect area well, resulting in tissue gap and inferior healing. Therefore, more morphological modification of DBM are required in cartilage tissue engineering.

#### 4.1.4 Chitosan

Chitosan is a deacetylated polysaccharide material originating from chitin. The surface of chitosan is hydrophilic and can promote cell adhesion, proliferation, and differentiation. In addition, the positive charge of the surface exhibits antibacterial activity and good biocompatibility. In 2000, Suhjk proposed that chitosan-based implants cause little allogeneic Immune Responses and fiber wrapping (Suh and Matthew, 2000). Jia *et al.* encapsulated rabbit synovial mesenchymal stem cells (SMSCs) in injectable chitosan hydrogels and implanted them into rabbit femoral cartilage defect model. Results showed that cartilage repair in the experimental group was significantly strengthened (Jia et al., 2019). Fang et al. (2014) co-cultured chondrocytes with porous poly (l-glutamic acid)/chitosan polyelectrolyte microspheres. Results showed that the microspheres improved the attachment and proliferation of chondrocytes. Experiments *in vivo* showed that chondrocytes/microsphere complex successfully repair cartilage tissue, making them an effective carrier for cartilage tissue engineering.

#### 4.1.5 Hyaluronic acid (HA)

HA, a linear macromolecular polysaccharide that widely distributed in human tissues and the ECM, is another widely used polysaccharide. HA can promote the migration, proliferation, and aggregation of bone cells, improve cell viscosity and support the survival of chondrocytes, so as to function in the generation of cartilage-bone. Therefore, HA and its derivative hydrogels are widely used as carriers for functional motifs (Park et al., 2019).

#### 4.1.6 Calcium alginate

Calcium alginate is a polysaccharide rich in guluronic acid and mannuronic acid. The advantages of adequate sources, low cost and low toxicity, as well as good absorbability, injectability, and biocompatibility attract the attention of researchers in drug delivery studies. Calcium alginate hydrogel has a large surface area and many internal pores, which is conducive to cell adhesion and material exchange. In 1989, Guo *et al.* firstly 3D-cultured chondrocytes in calcium alginate and observed that chondrocytes steadily proliferate and secrete the cartilage matrix (Guo et al., 1989). In 1995, Paige et al. (1995) subcutaneously implanted chondrocyte/calcium alginate complex into the backs of rats. After 6 weeks, ectopic hyaline cartilage was found (Paige et al., 1995). In 2002, Alsberg et al. (2002) injected chondrocyte/RGD/calcium alginate mixture into the backs of rats and detected ectopic cartilage formation.

### 4.2 Artificial synthetic materials

Synthetic material refers to artificially produced micromolecular monomers. Through chemical crosslinking, casting mold or 3D printing technology, the monomers assembled into macromolecular polymer materials. With excellent biocompatibility, highly standardized properties, mass production capacity, modification potential, and low immunogenicity, synthetic materials exhibit great potential as carriers in tissue engineering and regenerative medicine.

#### 4.2.1 Polymer materials

Polycaprolactone (PCL), polylactic acid (PLA), and other synthetic polymer compounds exhibited good biocompatibility and biodegradability, but their hydrophobic property limits the applications as carriers for hydrophilic peptides. Polyvinyl alcohol (PVA), polyethylene glycol (PEG), polyglycolic acid (PGA), polyethyleneimine (PEI) and other synthetic polymer compounds have good hydrophilicity, but are easy to degrade. PCL is widely used in biomedical research and has been approved for clinical application by the Food and Drug Administration (FDA) (Mkhabela and Ray, 2014). Malheiro et al. (2010) firstly reported the preparation of PCL/chitosan blend fibers and their application as scaffolds in tissue engineering in 2010. Poly(lactic-co-glycolic acid) (PLGA) is composed of lactic acid and glycolic acid. The biocompatibility and biodegradability of PLGA have also been approved by the FDA for clinical use. Kim et al. (2019) implanted fibrous PLGA scaffolds loaded with BMP7 and synovial mesenchymal stem cells into full-thickness rabbit cartilage defects, and realized higher secretion of proteoglycan and type II collagen, indicating better reconstruction of hyaline cartilage (Kim et al., 2019). In 2010, Wang et al. (2010) designed a composite structure of BMSCs/PDNA-TGF-β1/fibrin gel/PLGA sponge, and implanted it into cartilage defect area. After 12 weeks, the new cartilage came out and integrated well with the surrounding tissues.

#### 4.2.2 Micromolecular self-assembly materials

Some peptides can self-assemble by non-covalent interactions like hydrogen bonds, Van der Waals force and hydrophobic bonds. The assembling properties could be adjusted by modifying amino acid sequences and environmental parameters, so as to get nanostructures of various morphologies, including nanoribbons, nanotubes, spherical vesicles, nanofibers, nanowires, and ordered molecular chains (Smith et al., 2011). Self-assembly peptides exhibit various advantages of simple preparation, good biocompatibility, large functional surface, easy modification and excellent tissue permeability, therefore become a hotspot in cartilage tissue engineering (Knowles and Mezzenga, 2016). Previously, our team grafted TGF-β1-mimetic peptide, CM10, to self-assembling peptide hydrogel RADA16-1, and implanted it into full-thickness rabbit knee cartilage defect. Finally, effectively promoted chondrogenic differentiation of rabbit BMSCs *in vitro* and significant reconstruction of osteochondral units were found (Ye et al., 2022).Lu et al. (2018), Sun (Sun et al., 2018), Zhu et al. (2022) respectively grafted the sequences PFSSTKT and SKPPGTSS, derived from BMHP1, onto RADA16-1 hydrogel. Significantly strengthened recruitment of bone marrow stem cells (BMSCs) were found at the defect areas, according to their studies.

The temperature-responsive self-assembly of DNA opens up new space for the design of nanomaterials. Tetrahedral DNA nanostructures (TDNs) (Li et al., 2019), with advantages of small structure, blood circulation and simple preparation, gradually become good carriers of micromolecules. Contrary to self-assembling peptide, TDNs benefit from easier production and better thermostability. Moreover, TDNs showed excellent biocompatibility and biosafety compared to other inorganic nanomaterials (Chen et al., 2014). Li et al. (2011) grafted the bioactive nucleic acid molecule CpG to one chain of TDN by DNA hybridization, so as to introduce the functional fragment into macrophages through the endocytosis of TDN. Compared with the CpG monomer, the immune stimulation effect of functional TDN was significantly improved (Li et al., 2011). Lee et al. (2012) used TDN as an siRNA carrier and connected it with targeting molecule, folic acid, then successfully delivered siRNA to the solid tumor site of mice. After grafted onto TDN, siRNA showed significantly prolonged half-life *in vivo*, compared to monomer siRNA.

## 5 Modifications on functional motifs

### 5.1 Non-covalent bonding

Non-covalent bonding mainly involves van der Waals forces, hydrophobic bonds, hydrogen bonds, and charge distribution. Non-covalent binding immobilizes active peptides on the surface of the material, mainly through physical adsorption force, so as to promote cell adhesion, proliferation, and differentiation. Simple dispersal is commonly used for physical adsorption. Kantlehner *et al.* physically immobilized RGD on titanium surface and achieved improved adhesion of osteoblasts (Kantlehner et al., 2000; Mas-Moruno et al., 2014). However, owing to the weak binding force, low adsorption rate, poor stability and repeatability, the application of non-covalent bonds is still limited.

### 5.2 Covalent bonding

Chemical coupling is a typical method of covalent bonding. After introducing active groups (such as -NH2, -OH, -COOH, and active hydrogen) on the surfaces of the carrier, the peptides reacts with carrier by crosslinking agents (CDI, APTES, PPY, and SMP) (Pallarola et al., 2014), leading to improved physical and chemical properties of peptides, such as stability and controlled release behavior (Zreiqat et al., 2003).

In cartilage tissue engineering, complicated techniques, such as layer-by-layer self-assembling technique, are often used to modify composite scaffold. The principle is that compounds are deposited alternately layer by layer, by interaction between monomers including the strong binding force of chemical bonds and the weak binding force of non-covalent bonds. In this way, the monomers of different layers can spontaneously form a film with stable properties and specific functions. Chua *et al.* used layer-by-layer self-assembly technology to prepare a HA/chitosan/PEM/titanium substrate, and chemically crosslink it with RGD, so as to improve the adhesion and proliferation of osteoblasts (Chua et al., 2008). Moreover, Yang *et al.* developed a polydopamine (PDA) coating layer under weak alkaline condition, which increased the adhesion of nerve growth factor (NGF) increased, and promoted the differentiation and proliferation of human neural stem cells (NSC) (Yang et al., 2012). This is based on the super-adhesive effect of the PDA coating, and the improved hydrophilicity and cell adhesion ensured the immobilization and bioactivity of peptides.

## 6 Advantages and disadvantages of functional motifs

In general, existing growth factor products have several disadvantages. 1) Natural growth factors originate from animal vectors, which suffers from medical ethics and immunogenicity. 2) The high cost and low output during preparation and purification. 3) Complicated structures, especially after multiple processing in different chemical and physical microenvironments. 4) Poor biological stability. Due to the short biological half-life (Di, 2015), growth factors are easily degraded and inactivated, and usually need to be applied in large dosages to achieve the therapeutic goals. These limits hindered the widespread use of growth factors. To solve this problem, functional motifs were developed, and have exhibit remarkable advantages. The characteristics of motifs and proteins are listed in Table 2.

**TABLE 2:** Characteristics of motifs and proteins.

	Motif	Protein
Sequence length	Short	Long
Synthesis technique	Solid phase synthesis/Flow chemistry, easy	Recombinant protein expression in *E. coli*, complicated
Immunogenicity	Weak	Strong
Stability	Easier degradation and dilution	Relatively stabler
Modification	Easy modification for ending blocking and anti-degradation	Complicated modification of anti-degradation

### 6.1 Advantages of functional motifs

Synthetic peptides are superior to natural proteins in some ways. Firstly, functional motifs have good physical and chemical properties. 1) Functional motifs are peptide segments composed of amino acids, with simple structure and easy to adjust. 2) Functional motif can be loaded into different carriers by various methods, while maintained biological activity and stability. Zhang *et al.* added an extra cysteine residue to the C-terminus of CM10, which promoted the coupling of CM10 to the carrier, thereby improving the stability and function time of CM10 (Zhang et al., 2015a). Secondly, the functional motif has excellent biological properties. 1) The production of motifs by solid-phase synthesis does not require animal vectors. The low immunogenicity and no ethical issues make it possible in clinical application. 2) Excellent biocompatibility and biodegradability in natural physiological environments (Tiwari et al., 2012). 3) Potential targeting performance. Liposomes that bound with RGD peptide had been reported to exhibit targeting behaviors (Dharap et al., 2003). In a word, functional motifs can be directly synthesized in large quantities for their simple structures and low production costs. Therefore, functional motifs are increasingly popular in tissue engineering and regenerative medicine (Sekiya et al., 2002).

### 6.2 Disadvantages of functional motifs

However, functional motifs also face some shortcomings in terms of efficacy and physical properties. As a polymer of amino acids, peptides may be inactivated for chemical degradations and physical changes under complicated environments. Moreover, some functional motifs show limited efficiency when compared with cytokines. For instance, Renner *et al.* supplemented TGF-β1- related motifs, CM1 and CM2, to culture hMSCs, and the results showed that cell pellets only produced significantly lower GAG compared with TGF-β1 positive controls (12%–13% for CM1 and 7% for CM2) (Renner and Liu, 2013).

## 7 Research prospects of functional motifs in cartilage regeneration

Functional motifs have attracted extensive attention in the field of cartilage tissue engineering, owing to their simple structure, tunability, diverse functions, and low cost. Through reasonable design of microscopic structures and biological groups, peptide motifs can form nanostructures with specific morphologies and functions, which would make difference in chondrogenic researches. Future explorations on functional motifs should focus on the following aspects.

### 7.1 Biostability of functional motifs

Compared to macromolecular proteins, short peptide sequences are shorter and more easily to degrade by various proteases in organisms. In the future, short peptide molecules should be designed and synthesized, and the sequences should be modified to obtain more stable. For example, the stability of functional motif can be improved by ending blocking of acetylation and amidation, as well as blocking of ubiquitin modification sites.

### 7.2 Biological activity of functional motifs

Specific peptides of short sequence may only mimic partial structures of the functional domains. Thus, the administration dosages of short peptides are usually higher than that of whole proteins. Therefore, further researches are required to improve the simulation efficiency of biological activity with shorter sequences.

### 7.3 Self-assembly of functional motifs

Peptides may form granular, tubular, radial, fibrous mesh, and other specific configurations by self-assembling, which can improve the adhesion and integration with defect areas. However, the microstructure also affects the bioactivity. How to obtain a balance between structure and function is a challenge in future researchers.

### 7.4 Application prospect of functional motifs

Short peptides and their carrier scaffolds remain the hotspots in tissue engineering researches and clinical practice. Application researches should be conducted based on the following aspects.

Firstly, the interface between cartilage and subchondral bone is calcification layer, which bears the mechanical stress of defect area. The interface layer is convex to the cartilage, leading to stress concentration and mechanical load. In future studies, we should focus on the mechanism and growth factors involved in formation of calcification interface.

In addition, endochondral ossification shares similar biological processes with cartilage degeneration and osteoarthritis progression, including chondrocyte hypertrophy, apoptosis, and degradation of the cartilage matrix. It is vital to explore that whether peptide motifs participated in the processes, and how to regulate the cartilage matrix absorption and chondrogenic regeneration.

Finally, previous studies based on the biological function of scaffold on cells or organs, but how does motifs and factors exert influence on scaffold materials need to be studied. The morphology and content of regenerative biomaterials influences the repair effect. For example, physiological regulation of pH can be used for scaffold shaping, such as self-assembly peptides, HA, and thermosensitive hydrogels, to adapt to the morphology of defect area. In addition, appropriate degradation of active groups in composite scaffolds make it possible to exert specific bioactivity at different time points.

## Glossary

FGF2: fibroblast growth factor-2;
TGF-β: transforming growth factor-β;
PDGF: platelet-derived growth factor;
HGF: hematopoietic growth factor;
VEGF: vascular endothelial growth factor;
OA: Osteoarthritis;
RGD: ARG-Gly-ASP;
TPO: thrombopoietin;
TNF: tumor necrosis factor;
BMSC: bone marrow stem cell;
IGFBP: insulin-like growth factors-binding protein;
IGF: insulin-like growth factor;
bFGF: basic fibroblast growth factor;
BMHP: bone marrow homing peptide;
BMP: bone morphogenetic protein;
N-cadherin: Neurogenic cadherin;
HAV: His-Ala-Val;
CM: cytomodulin;
FNF-HMS: functional nanofi brous hollow microspheres;
hMSC: human mesenchymal stem cell;
hPLSC: human periodontal ligament stem cell;
GAG: glycosaminoglycan;
3D: three-dimensional;
DCM: decellularized cartilage matrix;
CT: computed tomography;
HGP: hydrogel particle;
ECM: extracellular matrix;
PEG: polyethylene glycol;
hPDC: human periosteum-derived cell;
Au-NP: gold nanoparticle;
PTHrP: parathyroid hormone-associated protein;
ADSC: adipose mesenchymal stem cell;
DBM: demineralized bone matrix;
SMSCs: synovial mesenchymal stem cells;
HA: hyaluronic acid;
PCL: polycaprolactone;
PLA: polylactic acid;
PVA: polyvinyl alcohol;
PEG: polyethylene glycol;
PGA: polyglycolic acid;
PEI: polyethyleneimine;
FDA: Food and Drug Administration;
PLGA: Poly(lactic-co-glycolic acid);
TDN: tetrahedral DNA nanostructure;
PDA: polydopamine;
NGF: nerve growth factor;
NSC: neural stem cell.
